# Impact of protein supplementation on semen quality, fertility, and *BMP1R* gene expression in sheep of Bangladesh

**DOI:** 10.1093/tas/txac072

**Published:** 2022-06-03

**Authors:** Md. Kabirul Islam Khan, Md. Iqbal Hossain, Md. Moksedul Momin

**Affiliations:** Department of Genetics and Animal Breeding, Chattogram Veterinary and Animal Sciences University, Khulshi, Chattogram 4225, Bangladesh; Department of Livestock Services, Upazila Livestock Office, Rangunia, Chattogram 4360, Bangladesh; Department of Genetics and Animal Breeding, Chattogram Veterinary and Animal Sciences University, Khulshi, Chattogram 4225, Bangladesh

**Keywords:** fertility, gene, protein supplements, sheep, traits

## Abstract

A study was carried out to know the impact of protein supplementation on fertility and expressions of the fertility gene *BMP1R*. Three International Organization for Standardization (ISO), isocaloric but different levels of protein supplement ration (11.70% crude protein [CP] for control/To, 12.99% CP for T1, and 13.86% CP for T2) were fed to three different groups of sheep. DNA was extracted from the whole blood sample for polymerase chain reaction (PCR) of the *BMP1R* fertility gene, and purified PCR products were sequenced by a Sanger sequencer. Sequence alignment, pair, and multi-alignment comparison of the *BMP1R* gene of the species were done with MEGA6. The semen volume (1.0 mL), sperm counts (4.2 × 10^7^ million), and percentage of normal (94.3%) and viable sperm (3.7%) were higher in treatment 2 than in the other two groups. The semen volume (1.0 mL), sperm counts (4.2 × 107 million), and the percentage of normal (94.3%) and viable sperm (3.7%) were higher in treatment 2 than in the other two groups. Ewes treated with supplemented, protein concentrate reached the conception at an earlier age (treatment 1, 9.5 ± 0.16 mo and treatment 2, 10.3 ± 0.04 mo) than control (9.8 ± 0.15 mo). The lambing interval varied, from 198 to 202 d. Lamb’s birth weights in three treated groups were ranging from 1.2 to 1.39 kg. The designated sequences of *BMP1R* gene revealed 100% homology with the sequence of Kazakh sheep. The present study indicated that the influence of nutrition on reproductive performance and genomic study will be helpful for the genetic improvement of low-productive sheep.

## INTRODUCTION

The expressions of the full genetic potential of an animal require improved ration, health care, and management. Among them, nutrition is the main component, especially protein. The quality of the protein in feed depends on both amino acid profile and its digestibility. In the ruminant, dietary protein can be classified as either rumen degradable protein or rumen un-degradable protein. The protein requirement of an animal depends on its physiological status and level of production.

Semen is the secretion of the male reproductive organ, containing the gametes (spermatozoa) and seminal fluids produced in the testis (Hafez and Hafez, 2013). The quality of semen plays a major role in determining the fertility and reproductive efficiency of animals. There are a number of studies such as Brown (1994) and Rekik et al. (2007), who conducted research on the effect of proteins on reproductive parameters in ram. Fernández et al. (2005) reported that appropriate nutritional management is essential for successful mating and conception in sheep flocks.

Genes *BMP15 and BMP1R* are related to ovulation rate and litter size that affect female fecundity in sheep (Barzegari et al., 2010; Javanmard et al., 2011). The *BMP15* gene is responsible for a cellular behavior, including the development and maturation of the oocytes (Hanrahan et al., 2004; Javanmard et al., 2011), and the *BMP1R* gene is responsible for fecundity or litter size in sheep.

The PCR, PCR-restriction fragment length polymorphism (RFLP), Sanger sequencer, RNA sequencing analyzer, next-generation sequencer, random amplified polymorphic DNA (RAPD), and some DNA-based technique are used on the animal fertility traits and reproductive performance in animals. Gene (*GDF9*, *BMP15*, and *BMP1R*) identification has been widely used in domestic animals, including sheep’s litter size study (Barzegari et al., 2010) and ovulation (Hanrahan et al., 2004). However, no in-depth study has yet been done on the semen quality, fertility, and gene related to fertility traits in sheep of Bangladesh using protein supplements in ration. Therefore, the study was carried out with the objectives 1) to know the effects of protein supplements on fertility traits in sheep and 2) to be familiar with genes responsible for fertility traits in sheep. 

## MATERIALS AND METHODS

An experiment was conducted in the rural areas of Chattogram district and Poultry Research and Training Centre (PRTC) laboratory of Chattogram Veterinary and Animal Sciences University (CVASU), Bangladesh, from January 2019 to March 2020 following the animal ethics rule and the ethics committee of CVASU (Memo no. CVASU/Dir (R&E) EC/2015/1011; date: December 27, 2018). 

### Animal Selection and Management

The phenotypic and morphological features of the sheep were recorded. Ninety-six indigenous sheep of Bangladesh were selected on the basis of their body condition score, health status, and normal clinical condition and age (8 to 9 mo). Then, six different farms were randomly divided into three treatment groups according to the location of farm, and each treatment contains 32 sheep (4 rams and 28 ewes). The three groups of sheep were reared under a semi-intensive system, and sheep were kept in a separate pen and fed them individually during the experimental period. They were observed regularly for any abnormalities, and at regular intervals, deworming was administered by the researchers.

### Experimental Ration Formulation and Design

Three ISO-caloric rations (12.00 MJ/kg dry matter [DM] metabolizable energy) containing different levels of protein were formulated (11.70% crude protein [CP] for control/To, 12.99% CP for T1, and 13.86% CP for T2) using available feed stuffs (Table 1), and concentrate mixture was supplied (0.250 kg/d/sheep) to the sheep up to the end of the study. Protein concentrate was provided in both T1 and T2 groups of sheep, but there was no protein concentrate supplied to the control group. All three groups of the sheep grazed for 5 to 7 h for a day in the natural pastures and fallow land.

**Table 1.** Ingredients and chemical composition of experimental ratio

Ingredients (kg)	Maize	Rice polish	Wheat bran	Soybean oil	Soybean meal	Protein concentrate	Dicalcium phosphate	DL-Methionine	Vitamin B premix	Common salt	Total amount	Calculated chemicals composition			
												Total CP, %	Total crude fiber, %	Ether extract, %	Total energy, MJ/Kg
Treatment 1 (T1)	57.5	21	10	0.75	5.0	2.5	1.15	0.6	1.0	0.5	100	12.99	4.45	6.18	12.00
Treatment 2 (T2)	55.5	21	10	0.75	5.0	4.5	1.15	0.6	1.0	0.5	100	13.86	4.49	6.31	12.00
Control (T0)	60.0	21	10	0.75	5.0	0	1.15	0.6	1.0	0.5	100	11.70	4.45	6.04	12.00

### Semen Collection and Evaluation

In the mid of the trial, six rams’ fertility was tested. Scrotal circumference (SC) was recorded by the spermatic cord grasping in the first week until the end of experiment in centimeters to find out the correlation with semen volume. The rams were trained for semen collection by the artificial vagina method (Matthews et al., 2003). During the trial period, semen collection was performed several times to assess sperm quality (physical and microscopic tests) following the procedure described by Santolaria et al. (2015) and Hossain et al. (2018).

### Record Keeping for the Fertility Traits in the Experimental Animals

Data were recorded for different fertility traits (e.g., estrus period, age at first lambing, gestation period, lambing interval, and lamb weight) of the experimental sheep up to the end of the experimental period. 

### Reproductive Traits of Ewes

Pregnancy was determined by abdominal palpation (Pratt and Hopkins, 1975), but it was confirmed from the record of mating date. Gestation length was determined from the day of service to the day of onset of labor. Pregnancy rate, lambing rate, litter size, and lamb survival rate were calculated using the formula described by Landais and Cissoko (1986) as follows:


$$\begin{array}{l} Conception\;rate\;(\%)=\frac{Number\;of\;ewes\;pregnant}{Number\;of\;ewes\;mated\;with\;rams}\times 100 \end{array}$$

$$\begin{array}{l} Lambing\;rate\;(\%)\;=\frac{Number\;of\;ewes\;lambing}{Number\;of\;ewes\;mated}\times 100 \end{array}$$

$$\begin{array}{l} Litter\;size\;(\%)=\frac{Number\;of\;lambs\;born}{Number\;of\;ewes\;lambed}\times 100 \end{array}$$

$$\begin{array}{l} Lamb\;survival\;rate\;(\%)=\frac{Number\;of\;offspring\;weaned}{Number\;of\;offspring\;produced}\times 100 \end{array}$$

### Blood Collection and DNA Extraction

A total of 40 blood samples (T1 = 16, T2 = 10, and T3 = 14) were tested for the presence of *BMP1R* genes whose amplicon sizes were 190 bp. Among all samples, 36 samples (T0 = 14, T2 = 10, and T0 = 12), 90%, were positive for *BMP1R* gene and 4 sample was negative due to damage. The DNA of the collected blood samples was extracted by the FAVORGEN BIOTECH CORP and FavorPrepTM blood genomic DNA extraction mini kit. DNA was then stored at −20 °C in a refrigerator before performing a polymerase chain reaction (PCR).

### PCR and Sequencing

Primers for PCR of *BMP1R* gene were cited from Hanrahan et al. (2004) and Maitra et al. (2016). Exon 1 of the *BMP1R* gene was then amplified using the following PCR primers (forward primer: 5ʹ-CCA GAG GAC AAT AGC AAA GCA AA-3ʹ, reverse primer: R-5ʹ-CAA GAT GTT TTC ATG CCT CAT CAA CAC GGT C-3ʹ with a resulting product size of 190 bp. PCR was carried out in a final reaction volume of 25 μL on an i-cycler (BIO-RAD, USA). PCR mixture consisted of 50 to 100 ng of genomic DNA, 200 μM of each deoxynucleotide triphosphates (dNTPs), 50 pM of each primer, 0.5 units of TaqDNA polymerase, and Taqbuffer having 1.5 mM MgCl_2_ for each reaction. The PCR cycle was accomplished by denaturation for 1 min at 94 °C; 35 cycles of denaturation at 94 °C for 45 s, annealing time with 53 °C temperatures for *BMP1R* gene, and extension step at 72 °C for 45 s with a final extension, at 72 °C for 5 min. The PCR products were visualized following electrophoresis through 1.8% ethidium bromide-stained agarose gel, and the fragments were photographed under Gel documentation Unit, and their sizes were estimated using a 100-bp DNA ladder (Fermentas International, Inc.). 

The post-PCR reaction product (5 μL) was cleaned with 2 μL of ExoSAP-IT (enzyme: ExoASP-IT). Then, it was incubated at 37 °C for 15 min for degrading the remaining primers and nucleotides. Finally, to inactivate the ExoSAP-IT enzymatic reaction, a mixed sample was incubated at 80 °C for 15 min (Affymetrix Pte Ltd, Singapore). Out of 36 positive samples, 5 best-purified PCR products ((T0 = 01, T1 = 02, and T2 = 02) were Sanger-sequenced with a big dye terminator v3.1 sequencing kit, and a 3730XL automated sequencer (Applied Biosystems, Foster City, CA) was used for purification of the PCR product. Then, nucleotide sequences were determined on both strands/reaction of PCR amplification products at the Macrogen sequencing facility (Macrogen Inc., Seoul, Korea) using ABI PRISM 3730XL Analyzer (96 capillary type).

### Statistical Analysis

The recorded data for the semen motility, live-dead ratio, number of ewes conceived, and pregnancy rate per sub-group were analyzed by using a completely randomized design. The collected data were analyzed by using the statistical package SAS (SAS, 2008). The least significant difference was used to compare the mean differences at the 5% level of significance. The nucleotide sequences were analyzed to determine the sequence alignment, and the neighbor-joining method by MEGA6 software was used for the phylogenic tree (Tamura et al., 2013).

## RESULTS AND DISCUSSION

### Seminal Traits

The mean and SEM values of different seminal traits are presented in Table 2, where differences in semen volume were not statistically significant (*P* > 0.05) among the three treatments. The semen volume was higher (1.0 mL) in treatment 2 than in the other two groups (0.8 and 0.9 mL). The present observation was similar to the findings of Fernández et al. (2005) and Kheradmand et al. (2006), which indicated that the control group produced less volume of semen than the treatment groups. Jibril et al. (2011) stated that semen volume was not influenced by the level of protein. There was no study found on the effect of protein supplementation on semen volume of ram in Bangladesh rather than the trails on reproductive performance (Pervage et al., 2009; Azizunnesa et al., 2013). The semen volume of ram per ejaculation in the present study was similar to the study of Pervage et al. (2009) and Azizunnesa et al. (2013), who obtained 0.86 to 1.0 and 1.2 to 1.5 mL, respectively, after feeding concentrate supplements. On the other hand, some researchers (e.g., Parker and Thwaites, 1972) reported that spermatogenesis in rams is affected by protein intake. The variation of semen volume in the present study may occur due to changes in protein percentages in the ration, age of the animal, and also the variation of farm management, and variation with other studies might be differences in protein intake and physiological conditions of the ram.

**Table 2.** Various seminal traits of ram

Traits	Control (*N* = 3)	Tratment 1 (*N* = 4)	Treatment 2 (*N* = 4)	SEM	*P*-value
Semen volume, mL	0.9^a^	0.8^a^	1.0^b^	0.03	0.03
Sperm count/10^7^ Million	3.9^a^	4.1^b^	4.2^b^	0.09	0.04
pH	7.2^a^	7.1^b^	6.9^b^	0.11	0.002
Scrotal diameter, cm	19.2	18.8	20.9	1.35	0.34

*N* is the number of rams in each treatment. Control = 11.70% CP, treatment 1 = 12.99% CP, and treatment 2 = 13.86% CP.

Means with different superscripts in the same row differ significantly (*P* < 0.05) among the treatment groups.

In the case of sperm count, treatment 2 contains 4.15 × 10^7^ million sperm, which was higher than the remaining two groups, that coincides with the findings of Jibril et al. (2011), who stated that increased CP intake (12%) above the minimum requirements resulted in the improved sperm concentration and favors spermatogenesis. In this current study, it was seen that the required level of protein increased the sperm concentrate. Similar results were obtained by Jibril et al. (2011) and Elmaz et al. (2007). However, Azizunnesa et al. (2013) reported more sperm count than the current study after feeding the concentrate supplements. Sperm production, as well as the total number of spermatozoa per milliliter, can be affected by the nature of the diet (Fernández et al., 2005), and extra protein lead to an increase in testicular size due to an increase in the volume and diameter of seminiferous tubules lead to higher sperm production (Abisaab et al., 1997; Hotzel et al., 1998).

Considering the pH (6.9 to 7.2), it was observed that there were significant (*P* < 0.002) differences among different groups. Jha et al. (2018) also measured the pH of fresh semen of ram ranging from 7.0 to 7.3. The variation among three groups of sheep in the present study occurred might be due to the variation in the chemical composition of ration, management system, age variation, and body configuration of rams. Similar factors were reported from a Nigerian study on Yankasa ram by Babashani (2015).

The scrotal diameter was more sizable voluminous in treatment 2 compared with other groups which were not statistically significant (*P* > 0.05). Scrotal diameter and testicular sizes were affected by nutrition, where testicular growth can be affected when animals are fed above their maintenance requirement (Murray et al., 1990). The voluminous size of the scrotal diameter of the treatment groups might be attributed to the optimum utilization of dietary protein at about 14% CP level as previously reported by Negesse et al. (2001). On the other hand, Azizunnesa et al. (2013) reported a similar SC from Bangladeshi indigenous ram after feeding the concentrate feed supplements with grazing in the natural pasture land.

### Sperm Morphology

Percentages of sperm morphology are presented in Table 3, which represents the percentage of normal sperm that is highly perceived in the case of treated groups (treatment 1, 92.1% and treatment 2, 94.3%) than in the control group. Here, 12.4% of sperm was abnormal in the case of control, which was higher than the other two groups. This outcome was consistent with the findings of Hossain et al. (2016), Kheradmand et al. (2006), and Jibril et al. (2011). It was revealed that sperm viability was not significant among the different treatments, but treatment group 2 showed 87.3% viable sperm, which was higher than the other two groups (Table 3) and agreed with the findings of Jibril et al. (2011) and Hossain et al. (2018), who observed that sperm viability in all groups was above 78% where the highest viability was recorded in the case of treatment groups than the control group, and there were no significant differences in sperm viability among the studied groups. The normal and abnormal and live and dead sperm cell are shown in Pictures 1 and 2, respectively.

**Table 3.** Sperm morphology of ram

Sperm morphology	Treatment			SEM	*P*-value
	Control (*N* = 3)	Treatment 1 (*N* = 4)	Treatment 2 (*N* = 4)		
Normal sperm cell, %	87.6^a^	92.1^ab^	94.3^b^	1.97	0.04
Abnormal sperm cell, %	12.4	7.9	5.7	1.97	0.04
Live sperm cell, %	79.8^a^	85.2^b^	86.9^b^	2.15	0.02
Dead sperm cell, %	20.2	14.8	13.1	2.15	0.02

*N* is the number of rams in each treatment. Control = 11.70% CP, treatment 1 = 12.99% CP, and treatment 2 = 13.86% CP.

Means with different superscripts in the same row differ significantly (*P* < 0.05) among the treatment groups.

**Picture 1.**
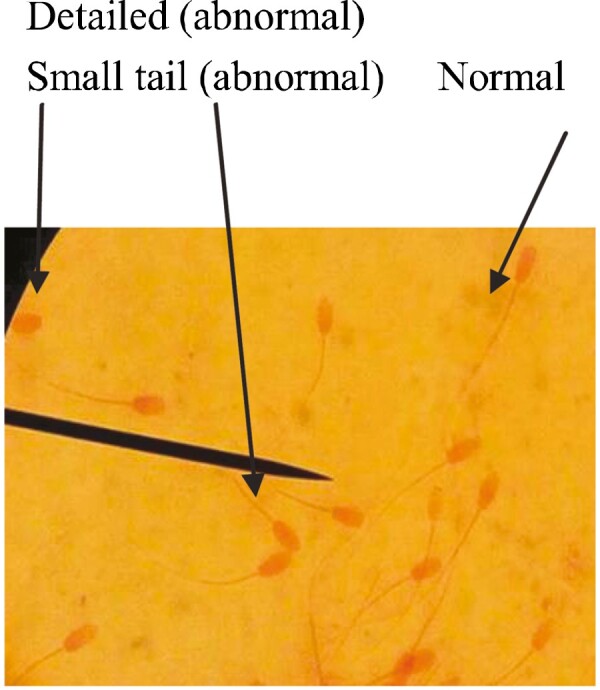
Normal and abnormal sperm.

**Picture 2.**
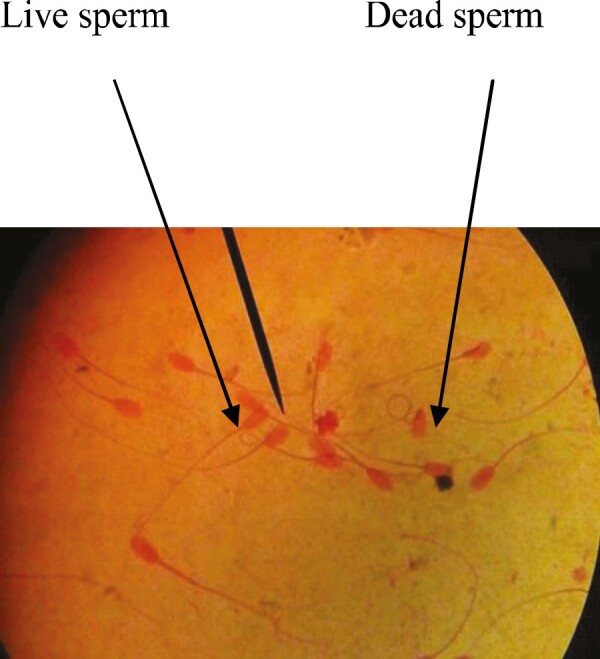
Live and dead sperm.

### Reproductive Parameters (Fertility Traits)

The various reproductive parameters (fertility traits) of indigenous ewes are presented in Table 4. The age at first conception varied with the differences in treatments. Ewes in the treated groups (protein concentrate) reached the conception at an earlier age (treatment 1, 10.3 mo and treatment 2, 9.8 mo) than in the control group (9.5 mo). Weight at first conception was significantly different (*P* < 0.05) among the three groups, where weight at the first conception was 11.8 kg in the case of treatment 2 group, which was higher than the other two groups. In the current study, the age at the first conception of ewes was higher than the study of Azizunnesa et al. (2013) and Al-Mansur et al. (2018) conducted in Bangladesh, but the weight at first conception was found similar. 

**Table 4.** Reproductive parameter (fertility traits) of indigenous ewes

Parameters	Control	Treatment 1	Treatment 2	*P*-value
Age at conception, mo	9.52^a^ ± 0.16 (22)	10.33^b^ ± 0.04 (24)	9.81^a^ ± 0.15 (24)	0.04
Weight at first conception, kg	10.8^a^ ± 0.29 (22)	12.3^c^ ± 0.18 (24)	11.8^b^ ± 0.24 (24)	0.14
Gestation period, d	152.3 ± 2.50 (22)	151.7 ± 2.10 (23)	149.0 ± 2.80 (24)	0.23
Lambing interval, d	198.2 ± 3.30 (20)	202.6 ± 5.20 (22)	198.6 ± 4.6 (22)	0.12
Lamb number, No.	1.10^a^ ± 0.13 (20)	1.50^b^ ± 0.16 (22)	1.70^b^ ± 0.15 (20)	0.05
Lamb birth weight, kg	1.20^a^ ± 0.04 (18)	1.30^a^ ± 0.03 (20)	1.70^b^ ± 0.04 (20)	0.07

Parenthesis indicates the number of observations. Control = 11.70% CP, treatment 1 = 12.99% CP, and treatment 2 = 13.86% CP.

Means with different superscripts in the same row differ significantly among the treatment groups (*P* < 0.05).

The gestation period (d) in the indigenous ewes varied from 149 to 152 d, and no significant differences were observed between treatments. However, a numerically longer (worse) value was observed in the control group (152.3 ± 2.50 d) compared with the treated groups. These findings were agreed with the findings of Rahman et al. (2011), Zohara et al. (2014), and AI-Mansur et al. (2018) and inconsistent with Sultana et al. (2011), who observed that the gestation period was longer in the treatment group than the control group. In the case of lambing interval (d), no significant differences were found between treatments, and these findings were similar to the results of Al-Mansur et al. (2018) and Hassan and Talukder (2012) and differed from the findings of Poonia (2008). Therefore, to minimize the lambing interval, post-lambing estrus interval and service per conception should be minimized.

The number of lambs per parturition (litter size) differed significantly (*P* < 0.05) between treatments, and more lambs per parturition were found in treatment 2 (1.7 ± 0.15 number). The higher litter size may be the result of an increased body weight gain of ewes fed a high energy diet during pre-mating and gestation period. The nutrition has ability to alter the liter size of ewes, as a rapid improvement in body weight gain is associated with an increase in ovulation rate and lambing rate (Zohara et al., 2014). The litter size of ewes was similar to the findings of Rahman et al. (2011) and Sultana et al. (2011). On the other hand, this finding was not supported by the work of Banerjee (2008), who showed that there was no variation in the case of lamb number among the various treated groups.

The birth weight of lambs in the treatment 2 group was 1.7 ± 0.04 kg, which was higher than the other two groups. The level of nutrition during the last weeks before parturition acted as a modifier for improving the birth weight of lambs. The birth weights of the lambs were also affected by the number of litter size (Duguma et al., 2012). The birth weight affects the lamb’s ability to ingest colostrum and receive proper mothering shortly after birth and thus to develop an ability to combat infections. This finding was harmonious with the results of Kabir et al. (2004), Rahman et al. (2011), and Zohara et al. (2014), who observed that the birth weight of lambs in the case of protein-treated groups was higher than the control group; this finding was not supported by Hassan and Talukder (2012), who said that there was no relation of birth weight of lamb with feed groups.

### Nucleotide Sequences of *BMP1R* Gene

**Figure 1.**
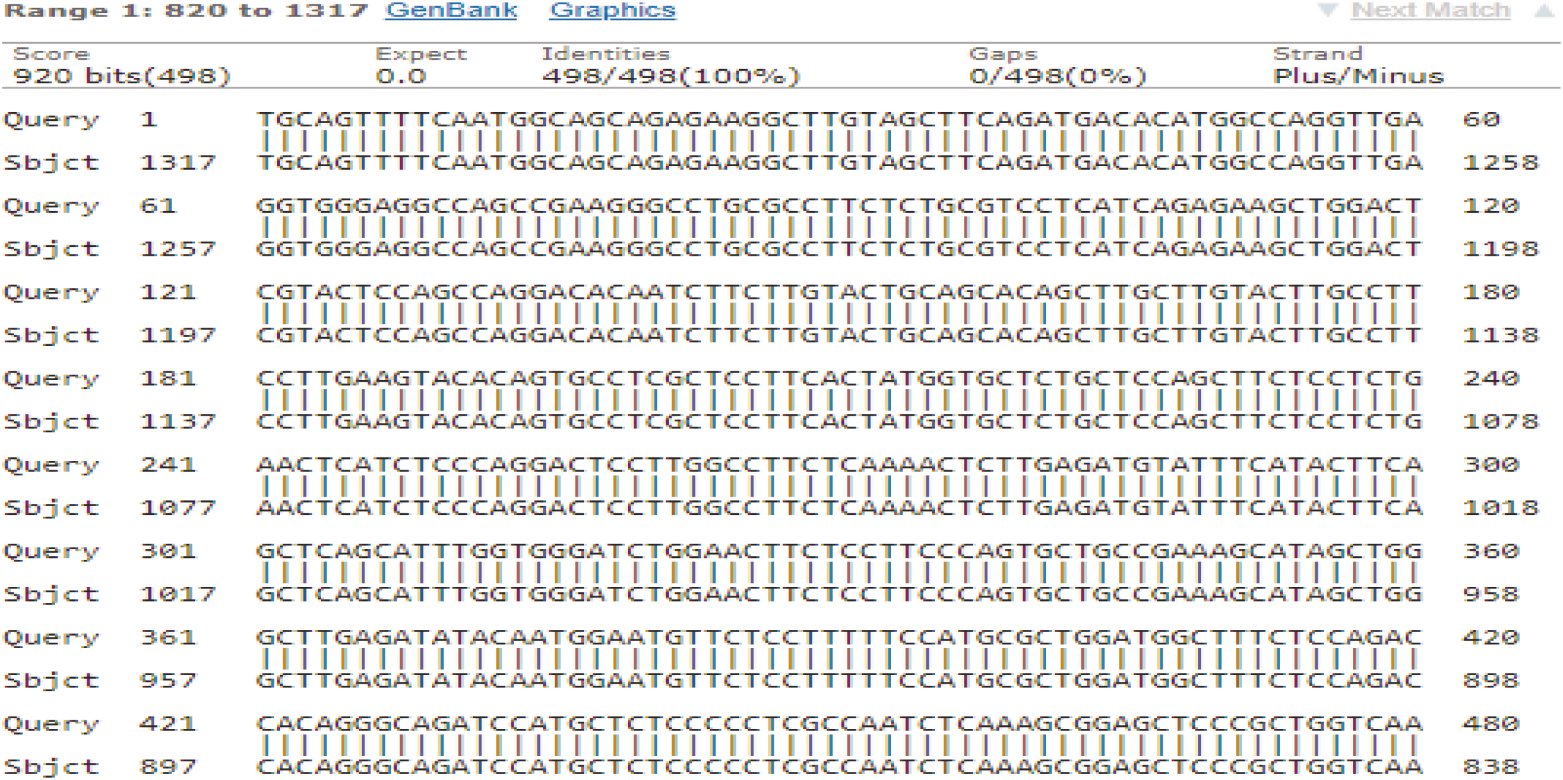
The 100% identity sequences of *Ovis aries* of *BMP1R* gene.

**Figure 2.**
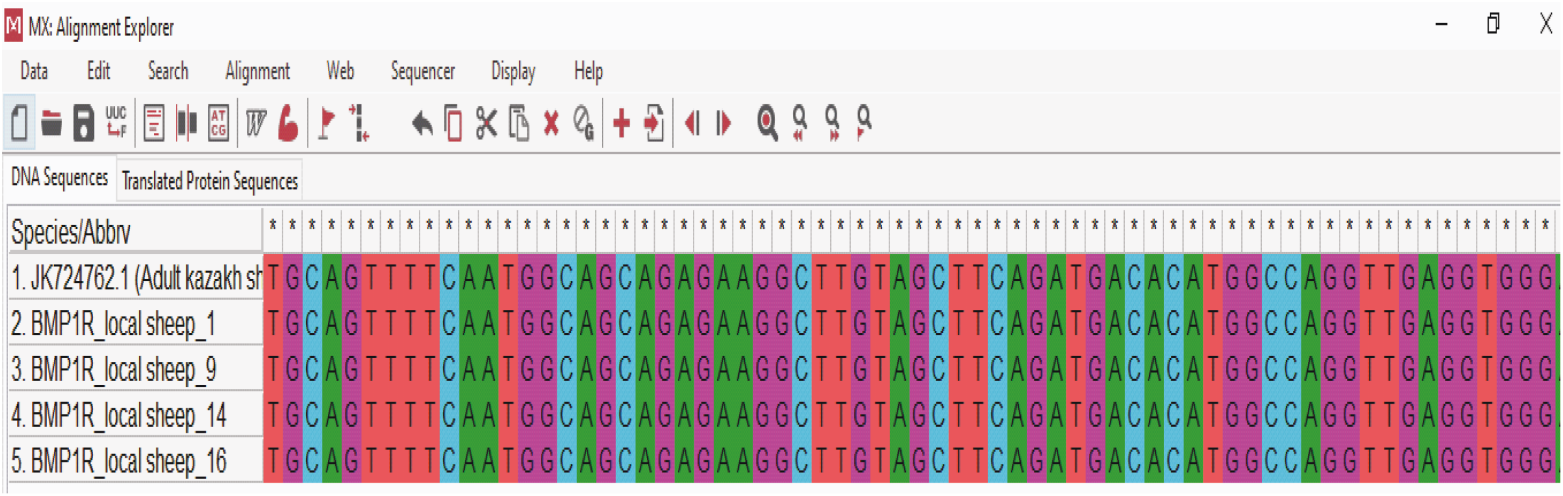
Nucleotide sequences alignment of *BMP1R* gene (e.g., Control = 1; treatment 1 = 2 and 3; and treatment 2 = 4 and 5).

The nucleotide sequence of the amplified fragment of the *BMP1R* gene of indigenous sheep and the reference nucleotide sequence were aligned using MEGA6. The alignment analysis demonstrated that the sequence of a *BMP1R* gene (Figures 1 and 2) was homologous with the sequence of Kazakh and some Indian breed of sheep. The designated sequence of the *BMP1R* gene of indigenous sheep showed 100% homogeneity with the Kazakh sheep (NCBI accession MZ848101-MZ848128) breed (Tarlykov et al., 2021). The evolutionary divergence among were from 0 to 1.10 that’s determined the sheep are most closely related to each other, which was supported by the findings of Tajima and Nei (1984) and Hossain et al. (2020).

### Evolutionary Relationship Taxa of *BMP1R* Gene

**Figure 3.**
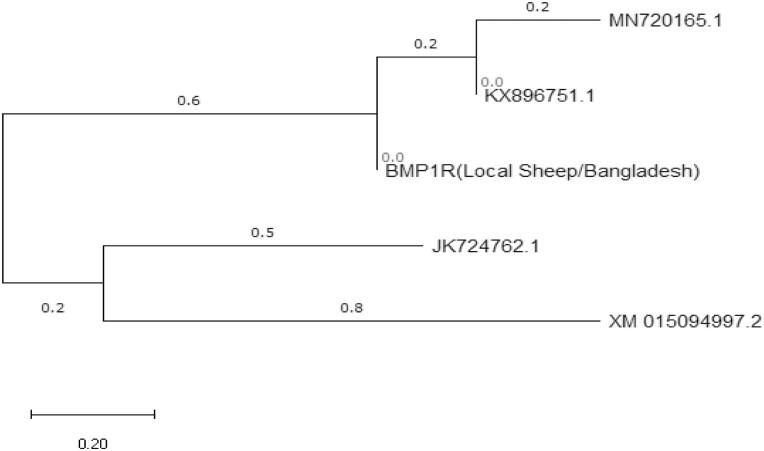
Neighbor-joining phylogenetic tree drawn based on nucleotide sequences of the *BMP1R* gene. Legends: MN720165.1 = *Ovis aries* (Rajasthan 304501, India)/(*BMPR1B*); KX896751.1 = *Ovis aries* (Chhotanagpuri sheep)/(FecB); JK724762.1 = *Ovis aries* (Kazakh sheep)/(*BMPR1B*) gene, complete cds; XM015094997.2 = *Ovis aries*/(FKBP4).

The phylogenetic tree was constructed using neighbor Maximum Composite Likelihood method, and the evolutionary history was inferred for *BMP1R* gene only. The branching pattern of the tree was used to determine the most closely related pairs of the sequence (Figure 3). Phylogenetic trees are important tools for organizing knowledge of biological diversity, and they communicate hypothesized evolutionary relationships among nested groups of taxa that are supported by sharing traits known as synapomorphies (Novick et al., 2011). Taxa that share a more recent common ancestor must be more closely related to each other than to another taxon with a less recent common ancestor (Dees et al., 2014). The branching pattern of the tree was used to determine the most closely related pairs of the sequence. Comparative studies of sequences were used in a wide range of taxonomic levels, to evaluate phylogenetic relationships. The phylogeny results of a study (Bibinu et al., 2016) based on nucleotide sequences of *BMPR1B* showed a similar clustering of sequences among the various breed of sheep those obtained in this study. The designated *BMP1R* sequences were closely related to the sequence of Chhotanagpuri sheep (KX896751.1) breed (Oraon et al., 2016) and other Indian sheep breeds (e.g., Malpura, Chokla, Deccani, and Nellore) indicated by Sawaimul et al. (2014) and Ramachandran et al. (2015). The phylogenetic tree in the present study indicated that the studies sheep population is closely related to Indian sheep breeds than other Asian sheep breeds. This close relation in gene sequences arose due to the same environmental conditions and ancestral relationship.

In conclusion, the studied sheep showed that the seminal traits were better in protein-rich feed group (13.9% CP) than the other two groups, which suggests that improving dietary intake above maintenance requirements had a positive impact on rams’ fertility. Similarly, protein supplemented feed (13.0% CP, 13.9% CP) showed the better reproductive performance of ewes. The alignment analysis demonstrated that the sequence of *BMP1R* gene was homologous to the sequence of Kazakh sheep, and the phylogenetic branching pattern showed the relatedness of the nucleotide sequence of the studied sheep with Indian sheep breed elsewhere. The potential role of all the possible mutations in *BMP1R* of fertility and ovulation rate in indigenous ewes needs further investigations, including DNA re-sequencing, complete gene sequencing, and molecular marker analysis. However, the current findings will be helpful in conserving the sheep and can be applied for the genomic improvement of low-productive sheep.
